# Designing Nostalgic Tangible User Interface Application for Elderly People

**DOI:** 10.1007/978-3-030-58805-2_56

**Published:** 2020-08-12

**Authors:** Way Kiat Bong, Florian Maußer, Margot van Eck, Diogo De Araujo, Jorg Tibosch, Tobias Glaum, Weiqin Chen

**Affiliations:** 8 Institute Integriert Studieren, JKU Linz, Linz, Austria; 9 Jack Baskin School of Engineering, UC Santa Cruz, Santa Cruz, CA USA; 10 Dipartimento di Meccanica, Politecnico di Milano, Milan, Italy; 11 Support Centre for Students with Special Needs, Masaryk University Brno, Brno, Czech Republic; OsloMet – Oslo Metropolitan University, Postboks 4, St. Olavs Plass, 0130 Oslo, Norway

**Keywords:** Tangible user interface, Nostalgia, Elderly people

## Abstract

Our elderly population faces challenges in accepting and using new digital technology, and tangible user interface (TUI) can contribute as a more intuitive user interface in addressing these challenges. Studies have shown that nostalgic memories trigger positive emotions, which can provide better experiences for elderly people in learning and using new technology. However, the use of nostalgia in TUI for elderly people has been little and therefore the understanding on how nostalgia can contribute in TUI promoting technology acceptance among elderly people is limited. In order to address this knowledge gap, in this study we have created a nostalgic TUI application for elderly people through three iterations of design, development and evaluation. The results show that by adopting the element of nostalgia into the TUI application, elderly people could learn to use new technology in a more intuitive way. They could relate the new technology to their old positive memories. However, they had expectations that the TUI application would work exactly like the old fashioned way. Through the research process, we gathered and reflected on the lessons learned, which can serve as guidelines for using the concept of nostalgia in designing TUI application for elderly people’s technology acceptance.

## Introduction

Recent studies have been focusing on improving elderly’s acceptance and use of information and communication technology (ICT). The use of ICT among elderly people can have positive impacts on their daily life, but many elderly people are not benefiting from it as they felt “intimidated” and “anxious” with the new technology [1]. Thus, while designing ICT for elderly people, it is crucial to ensure that ICT appears inviting, user-friendly and easy to use. Tangible user interface (TUI) allows the users to interact with the digital information using everyday physical objects [2], which can result in more intuitive and effortless use of ICT. It has therefore been identified as having the potential in addressing elderly people’s challenges in accepting and using new technology [3–5].

In order to achieve a design of TUI that is inviting, user-friendly and easy to use, the concept of nostalgia can be used. According to Sedikides and Wildschut [6], nostalgia is a self-relevant, most of the time positive than negative emotions that link one to his or her past. Therefore, it can help people in finding meaning in their lives by primarily increasing social connectedness, i.e. a sense of belongingness and acceptance, and secondarily by augmenting self-continuity (a sense of connection between one’s past and one’s present). Studies have shown that nostalgic memories can trigger positive memories [6, 7]. These positive emotions can contribute in providing better user experiences for elderly people in terms of learning and using new technology.

However, the use of nostalgia in TUI for elderly people has been little. Nilsson, Johansson [7] developed a nostalgic TUI prototype, named “Nostalgia”. It consisted a textile runner and an old fashioned radio placed on a table. By pressing on the runner, a choice would be made for listening to the news or music. Although the study indicated that using nostalgia could promote technology acceptance among elderly participants, Nilsson, Johansson [7] stated that their work focused more on the process of a participatory design instead of the understanding why Nostalgia gained good acceptance among them. In another similar works, Seo, Sungkajun [8] demonstrated the potential of their interactive plants among the elderly people living in an assisted living facility. However, the ways how the nostalgic TUI application could promote better technology acceptance was not clearly illustrated.

In this study we aim to investigate how nostalgia can contribute in designing TUI application for better technology acceptance among elderly people by designing and developing a nostalgic TUI application for them. While improving elderly’s technology acceptance, we hoped that they could have more social interaction through the use of TUI application. In this paper, through our research process, we gathered and reflected upon the lessons learned in using the concept of nostalgia in designing TUI for elderly people.

## Prototypes

The TUI application’s main functionality is to enable elderly people to develop new potential friendship by making calls to other users who had the same interest. It was developed through three iterations of design, development and evaluation. We involved two elderly participants (P1 and P2) to evaluate the prototype in each iteration. Both of them were above 70 years old and had been using ICT actively. They were recruited since they were interested and had experience working in helping other elderly people in using ICT. P2 even involved in giving talks and seminars to inspire elderly people to use ICT. By having them to evaluate the prototype, we hoped that their feedback could contribute to a better designed TUI application for elderly ICT users.

During the evaluation, the prototype was first presented to them. They were then interviewed to provide their feedback about the prototype. Questions related to the design of the prototype, for instance its size and weight, the way they would use the prototype and so forth were asked. Using the gathered feedback, we improved the design and further developed the prototype in the next iteration.

### Iterations of Design, Development and Evaluation

**Fig. 1.**
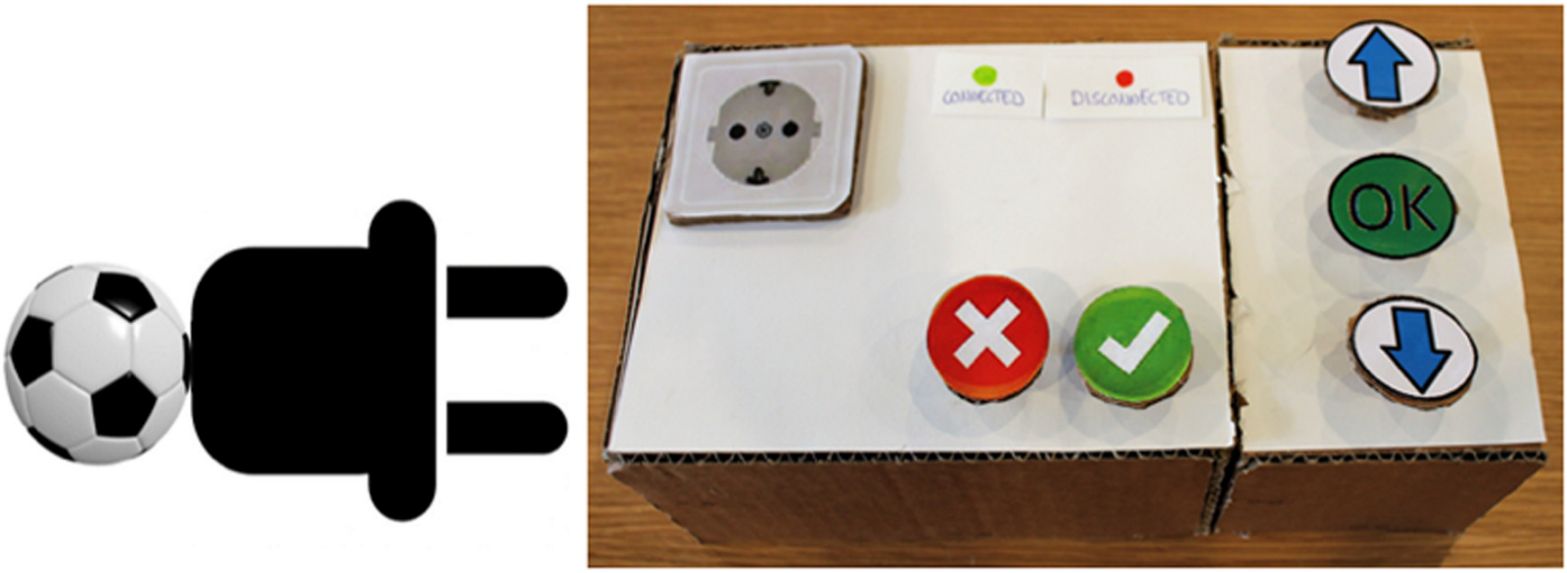
Plug and play paper prototype with interest plug (to the left) and console box (to the right)

In the first iteration, the group produced a low fidelity paper prototype that consisted of a box with plugs (Fig. 1). The idea was inspired by the old fashioned plug and play gaming console. A symbol object representing an interest was attached to a plug. For instance, a football representing sports is attached to a plug in Fig. 1. The user was expected to connect the ‘interest plug’ onto a socket on the console box to find other users with the same interest. The console box together with several interest plugs (sports, news, music, literature, travel, etc.) were presented to P1. During the evaluation, P1 had problem understanding the prototype. He commented that the plug and play gaming console was not familiar to him, since he was not interested in such games. After explaining to him, the evaluation continued. Despite the difficulty in understanding the design, he provided his opinion regarding the functionality, size, weight and overall design of the TUI prototype.

**Fig. 2.**
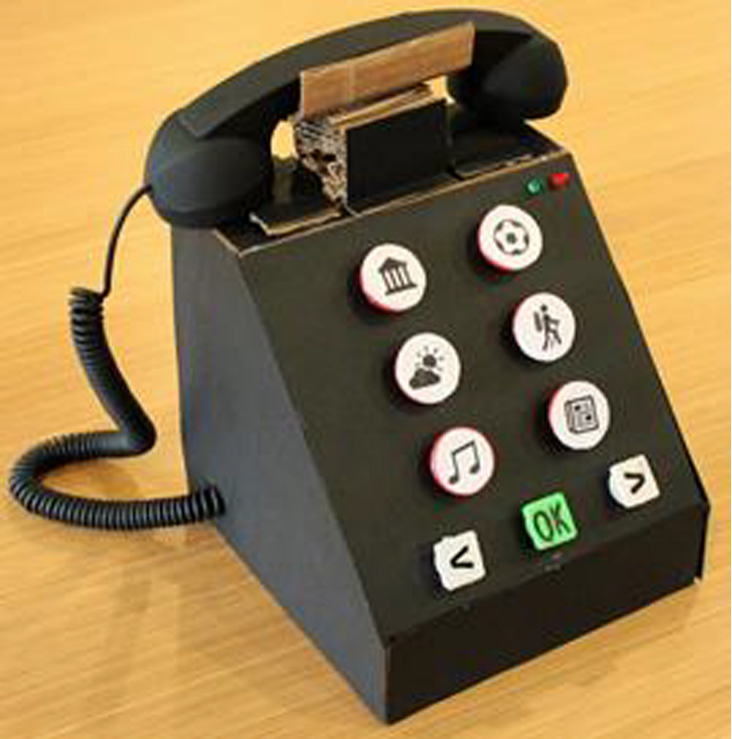
Old fashioned telephone paper prototype

Using the gathered feedback, the group decided to make another paper prototype in the second iteration so that they could compare the two designs. To make the nostalgic TUI application appeared more familiar to a larger group of users, the group decided to adopt the design of an old fashioned telephone. During the evaluation, we presented P2 the paper prototype of plug and play console (Fig. 1), and the paper prototype of old fashioned telephone (Fig. 2).

She was asked about her opinion about both prototypes. She preferred the telephone over the plug and play box. The telephone was found to be more familiar to her and therefore easier to understand. Same as P1, she provided her feedback about the functionality, size, weight and so forth, so that we could further improve the design of the TUI telephone in the next iteration.

### TUI Telephone

**Fig. 3.**
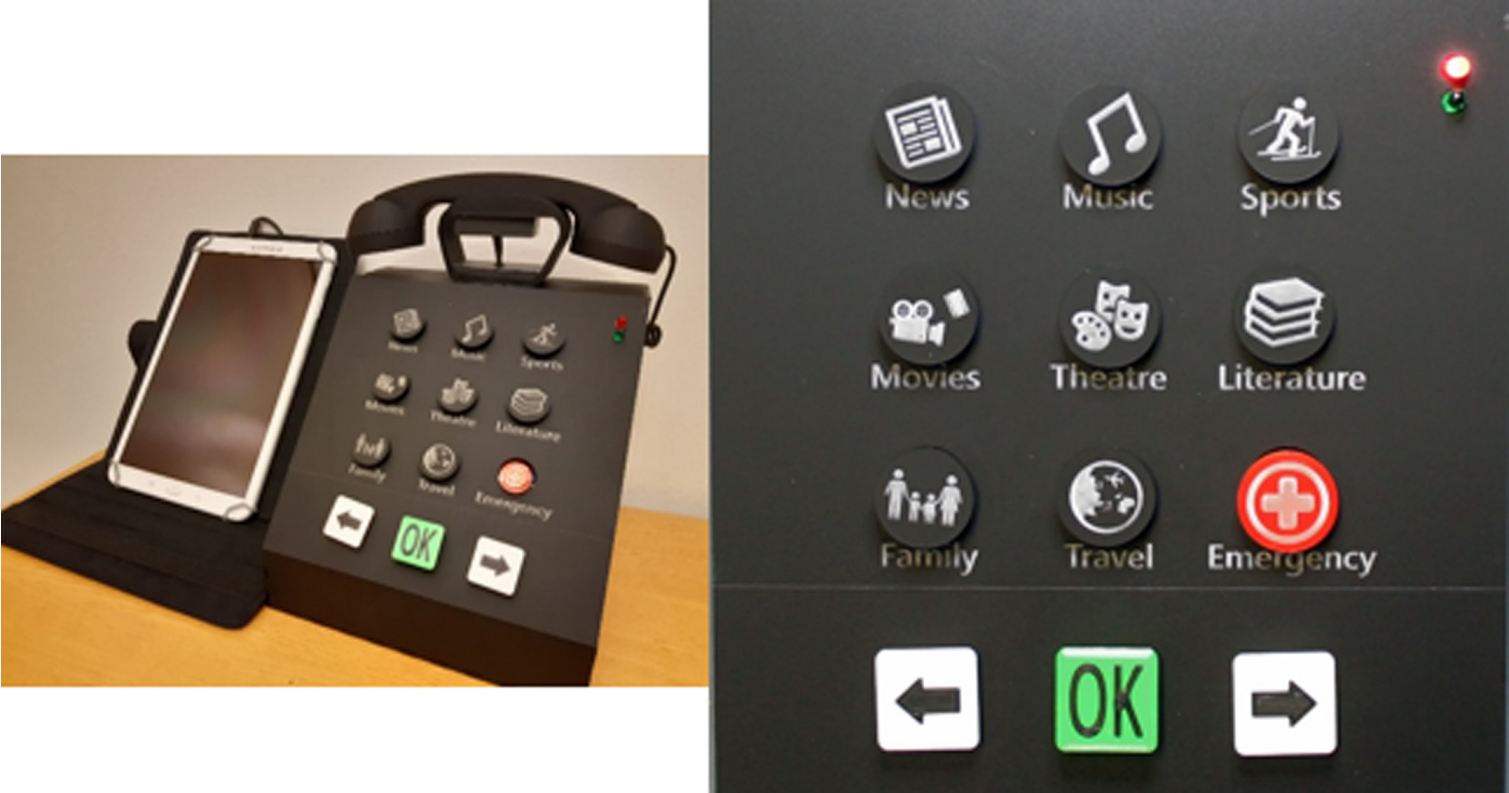
TUI telephone (to the left) and its 12-key keypad (to the right)

In the third iteration, a high fidelity prototype of the TUI telephone was created. As illustrated in Fig. 3, it consisted of an old fashioned TUI telephone (size 20 cm × 20 cm × 20 cm) connecting to a tablet. In the tablet there was a calling app installed. An Arduino board was embedded in the TUI telephone to inform the actions performed on the TUI telephone to the calling app in the tablet via Bluetooth.

There were 12 buttons on the TUI telephone, which was the same as the standard 12-key telephone keypad (10 digits, star sign and hash sign). The size was 3 cm × 3 cm for the square-shape buttons and 3 cm-diameter for the round buttons. Based on the feedback from the evaluation in the second iteration, for the design of 12-key TUI telephone keypad, we included eight interests (news, music, sports, movies, theatre, literature, family and travel), one button for emergency, two buttons indicating ‘Previous’ and ‘Next’ to navigate while selecting contacts on the tablet, and one ‘OK’ button to select a contact. There were a red light and a green light on the top right corner of the TUI telephone. When the users were making calls, the green light would turn on. The red light would flash when the call ended.

To use the TUI telephone, the users needed to pick up the handset and then press on a particular interest button. The action would trigger a list of online users who had the same interest displayed on the calling app in the tablet. They could then choose to make a call to one of the online users. In order to simulate nostalgic user experience, the users were also expected to use the handset while talking on the phone. The calls were limited to audio only without video, as we attempted to keep the functionality as simple as possible for the elderly users.

## Final Evaluation

### Participants and Tasks

Testing tasks were added into the evaluation in the third iteration. P1 who has involved in testing previous prototypes participated in the final evaluation. In addition, we set up a booth at a senior center and recruited five walk-in visitors (four females and one male) aged above 65 years old to participate in the final evaluation. All of them were ICT literate users and they performed the testing tasks individually at a time at the cafeteria at the senior center. All testers were asked to use the TUI telephone to make a call to someone based on their interests. They were observed while performing the testing tasks.

### Results

Both P1 and all five participants from the senior center liked and understood the design of the TUI telephone easily, as they could immediately relate the design of the TUI telephone to their old fashioned telephone. However, there were a few aspects that raised concerns among the participants.

First, they commented that the size of TUI telephone was too big. Most of them were concerned that the telephone might be too heavy to carry around for the other elderly people. On the other hand, it might then be suitable for elderly people who had special needs, for instance those who had visual impairment or problem with muscle control. For elderly people with visual impairment, the font size in the calling app had to be big, or they should be able to voice over correctly. The buttons on the keypad could be incorporated with braille as well. P1 suggested to provide options for the users to choose their own color and size for the telephone. Black color appeared to be slightly sad to him. Other usability issues included the color contrast on the buttons was not sufficient and the placement of the buttons and the labels was too close, which caused the shadow of the button to fall on the labels (refer Fig. 3 to the right).

In terms of the way of using the TUI telephone, some participants did not understand completely that the TUI telephone was used to interact with the calling app installed in the tablet. They thought the TUI telephone was a stand-alone device. In addition, they were confused as they were expecting that a telephone number would be needed to make a call. They wondered how they could dial the number when it only showed interests on the keypad. After explaining to them that the numbers were not required and they should use interest buttons to find other users, they understood the idea. We expected the participants to press an interest button first, then pick up the handset and choose an online contact to make a call. However, while conducting the testing tasks, the participants showed the other way around. After clarifying with them, we understood that they were used to picking up the phone first, only then they would press the number buttons to make a call.

All participants were willing to test and try out the TUI telephone because they were interested in new technology and used ICT very often. Since they were already experienced ICT users, they were unsure if the TUI telephone would be suitable for elderly ICT users like them. On the other hand, they agreed that it could reach out to their peers who were more skeptical towards new technology. The use of TUI telephone was commented more suitable to be use in a group setting than individual, home setting. They suggested that a group of elderly senior center visitor could use it together and call to elderly users at other senior centers. Lastly, they suggested more functionalities could be provided than just simply calling someone based on interests. For elderly people, they often needed to call to organizations providing services such as hospital, senior center, bank and so forth.

## Discussion

This study supports the findings from previous studies in designing nostalgic TUI application for elderly people [5, 7–9], i.e. the nostalgic TUI telephone in this study managed to gain positive user experience among elderly people. It appeared intuitive, user-friendly and inviting for them. Through the entire research process, we gathered and reflected upon our lessons learned which can serve as guidelines for using the concept of nostalgia in designing TUI application for elderly people’s technology acceptance.

First, nostalgia applies not only to the object, but also to the behavior. Our observation indicates that elderly people expected the TUI application to be used in the old fashioned way. For instance, they would pick up the handset first before pressing on any interest button. They also anticipated to dial telephone numbers and therefore had challenges understanding the concept of getting contact with users who had the same interest by pressing interest button. This finding is consistent of Li, Hu [10] who found out that the elderly users encountered difficulties when performing tasks which were different from their recalled memories, i.e. opening an incoming message using touch gesture. While designing a nostalgic TUI, or any nostalgic ICT application, this is an important factor to consider in terms of the elderly users’ interaction with the application.

Second, a more commonly shared nostalgia can reach more diverse elderly user group. Examples of commonly shared nostalgic object include jukebox designed for people with dementia [11] and slots-machine for elderly people [10]. The authors from these studies claimed that the chosen nostalgic object could enhance familiarity for the target users by employing metaphor and thus providing intuitive interaction. In our study, the feedback from the elderly participants indicate that the design of an old fashioned telephone, which is a more commonly shared nostalgia was preferred over a plug and play gaming console.

Third, Nostalgia is for the present and the future. While triggering elderly people’s positive emotions and old memories, it is important to remember that the design with nostalgia element shall aim for their present and future use, rather than leading them to be reluctant in accepting the new technology. Similar to Nilsson, Johansson [7] ’s study, our findings indicate that nostalgic TUI application managed to trigger the elderly people’s curiosity towards the new technology and we hope that such design could inspire them to be more open about their present and future ICT use.

Fourth, there will be gaps between the new technology and nostalgia, and it is important to reduce these gaps. It is difficult to have the new technology work exactly the same as the old fashioned way. The gap occurred when the elderly users’ expectation was different from how the TUI telephone actually worked. One example was the TUI telephone would not require the elderly users to dial the numbers to make a call. Similarly in Li, Hu [10] ’s study, the gap occurred when the incoming messages were in digital form and the users were not expecting to respond to them using touch gesture. It is important to take into consideration of these potential gaps when using the concept of Nostalgia in designing TUI application for elderly people. Such gaps can be addressed by providing facilitation and guided instructions. Li, Hu [10] suggested that paper instruction was necessary in order to provide text-based guidance to the elderly users.

We acknowledge that the design of TUI telephone was not completely TUI. By having the tablet as a display, the entire application was actually a combination of TUI and GUI (graphical user interface). However, the focus of the study was on designing and developing the TUI object, and we managed to identify the ways nostalgia can contribute in designing TUI application for better technology acceptance among elderly people. In addition, the use of mobile technology where the users are interacting with GUI has been heavily studied with the goal to address the special needs of elderly users in using these mobile technologies [12]. The future elderly ICT users might feel less “intimidated” and “anxious” with new technology. Combining TUI and GUI in a design of ICT application can be the trend for the future gerontechnology. The presented work might therefore serve as an inspiration for future studies that aim to achieve that.

Two major limitations in this study should be noted. First, limited number of elderly participants were involved in the process of design and evaluation of the prototypes. Second, they were not representatives of the target user group. The participants were experienced ICT users and had higher level ICT skills than target users of the prototype. The design of the prototype is at its preliminary stage and future development should address these limitations by involving more target users in the process.

## Conclusion and Future Work

In summary, the paper demonstrates the process of design, development and evaluation of a nostalgic TUI application for the technology acceptance among elderly people. A list of lessons learned have been gathered and presented, which could provide better understanding in using the concept of nostalgia in designing TUI application for elderly people’s technology acceptance. One of the implications of this study includes informing the researchers, designers and developers about the potential of adopting nostalgia in designing TUI or even general ICT tools for elderly users. Nostalgia triggers positive emotions and experiences of elderly people, and the nostalgic TUI application could therefore have a less “intimidating” and more inviting appearance for this user group. Such application could be potentially useful for people with dementia.

In the future, we hope to improve the design of the TUI telephone in two versions. First version is a TUI telephone with tablet while the other one is a purely TUI without the use of tablet. Both versions shall be further developed into fully functional prototypes. A comparative case study where both prototypes can be used and tested at senior centers by the elderly users will inform us more about the impacts of the nostalgic TUI telephone on elderly’s technology acceptance and social interaction, along with their preferences in terms of the design of nostalgic TUI application. Elderly users with diverse abilities, preferences, ICT skills and so forth will be included in the case study.
